# High-Resolution OCT Reveals Age-Associated Variation in the Region Posterior to the External Limiting Membrane

**DOI:** 10.1167/tvst.14.1.16

**Published:** 2025-01-15

**Authors:** Muhammad Usman Jamil, Jungeun Won, Stefan B. Ploner, Anna Marmalidou, Hiroyuki Takahashi, Stephanie Kaiser, Yunchan Hwang, Omar Abu-Qamar, Antonio Yaghy, Andre J. Witkin, Peter Y. Zhao, Shilpa Desai, Jay S. Duker, Andreas Maier, James G. Fujimoto, Nadia K. Waheed

**Affiliations:** 1 New England Eye Center, Tufts Medical Center, Boston, MA, USA; 2 Research Laboratory of Electronics and Department of Electrical Engineering and Computer Science, Massachusetts Institute of Technology, Cambridge, MA, USA; 3 Pattern Recognition Lab, Friedrich-Alexander-Universität Erlangen-Nürnberg, Erlangen, Germany

**Keywords:** external limiting membrane, inner segment/outer segment junction, photoreceptor inner segments, high-resolution OCT, motion correction, volume fusion

## Abstract

**Purpose:**

To evaluate visibility of a sub-band posterior to the external limiting membrane (ELM) and assess its age-associated variation.

**Methods:**

In a retrospective cross-sectional study, normal eyes were imaged using a high-resolution spectral-domain optical coherence tomography (SD-OCT) prototype (2.7-µm axial resolution). Volume fusion of six sequential scans (each 500 × 500 A-scans over 6 mm × 6 mm) was performed in the motion correction and volume reconstruction in OCT (MoReOCT) framework to enhance feature visibility in OCT. The subjects were divided into three groups: young (21–40 years old), middle (41–60 years old), and older (>60 years old). Three expert graders assessed the visibility of the sub-band on B-scans, and its A-scan intensity relative to ELM intensity (peak intensity ratio) was measured.

**Results:**

Forty-four eyes of 44 subjects were imaged. The sub-band, tentatively attributed to the photoreceptor myoid, can be visualized under high-resolution OCT. The B-scan gradings showed that sub-band visibility increased with age (visible in 16.7%, 47.2%, and 66.7% of the young, middle, and older age groups, respectively). The gradings were statistically different among age groups at 1 mm and 2 mm nasal and 1 mm and 2 mm temporal (*P* < 0.04) from the foveal center. Similarly, the mean peak intensity ratios of the sub-band to the ELM were 71.6%, 77.5%, and 85.2% in the young, middle, and older age groups, respectively, and were positively correlated with age at 1 mm temporal (*P* = 0.012) and 2 mm temporal (*P* < 0.001).

**Conclusions:**

High-resolution OCT, combined with advanced volume fusion, enables visualization of the photoreceptor myoid and investigation of its age-associated variations.

**Translational Relevance:**

Investigating the sub-band can advance our understanding of photoreceptors and their association with aging and disease pathogenesis.

## Introduction

Optical coherence tomography (OCT) is a powerful imaging modality that is routinely used to diagnose and manage ocular diseases as a standard of ophthalmic care.^1^^,^^2^ OCT allows precise measurements of structural alterations in different retinal layers, facilitating a better understanding of disease severity and progression. OCT has enabled visualization of multiple outer retinal bands, such as the external limiting membrane (ELM), ellipsoid zone (EZ), photoreceptor layer, retinal pigment epithelium (RPE) layer, and Bruch's membrane.^3^^–^^6^ The EZ is also referred to as the photoreceptor inner segment/outer segment (IS/OS) junction, and its clinical significance in retinal diseases, such as age-related macular degeneration and diabetic retinopathy, has been extensively studied.^7^^–^^10^

In the current OCT literature, the hyporeflective region between the ELM and IS/OS junction (or EZ) has been attributed to the myoid zone^5^ and does not exhibit hyperreflective features in normal eyes. The term myoid “zone” was chosen because of the challenges in identifying the structure that causes the absence of reflection.^5^ This region is less reflective because there are fewer mitochondria in the myoid than there are in the photoreceptor ellipsoid, except the foveola, where there are more mitochondria in the myoid.^5^

With recent advances in OCT technology, finer structures can be observed within the outer retina, providing insight into near cellular features and allowing more detailed analysis.^11^^–^^13^ For example, an anatomical correlation of the hyporeflective region between the ELM and IS/OS junction (or EZ) was investigated using a visible-light OCT prototype instrument with an axial resolution of 1.0 µm.^14^ The study showed that the myoid and inner ellipsoid portion of the photoreceptors can be visualized as a sub-band between the ELM and IS/OS junction in humans and mice using visible-light OCT.^14^^,^^15^ Although it achieves superior axial resolution, visible-light OCT is primarily available as a research prototype, limiting its use in clinics for larger patient enrollment.

In this study, we evaluated the sub-band posterior to the ELM and its association with age using a near-infrared (NIR) high-resolution OCT prototype instrument^16^ combined with advanced three-dimensional (3D) motion correction and volume fusion techniques.^17^^,^^18^ High-resolution spectral-domain OCT (SD-OCT) can achieve an axial resolution of ∼3 µm,^16^^,^^19^^,^^20^ compared to 5- to 7-µm axial resolutions of standard commercial SD-OCT instruments. We hypothesized that NIR high-resolution OCT can also visualize the sub-band, attributed to the myoid and inner ellipsoid zone, as seen under visible-light OCT. High-resolution NIR SD-OCT instruments are becoming more clinically accessible, and assessing the sub-band can provide a deeper understanding of photoreceptors and their associations with aging and disease pathogenesis.

## Methods

### Human Subject Imaging

This retrospective cross-sectional study included subjects recruited at the New England Eye Center at Tufts Medical Center (Boston, MA). All eyes were from healthy subjects or those having diabetes mellitus (DM) without clinically diagnosed retinopathy. Diabetic subjects underwent fundus examinations to confirm healthy macula. Subjects with severe cataracts were excluded. Written informed consent was obtained from all subjects prior to imaging, and procedures were compliant with the tenets of the Declaration of Helsinki. OCT images were acquired under research protocols approved by the Institutional Review Board at Tufts Medical Center and the Massachusetts Institute of Technology (MIT) Committee for the Use of Humans as Experimental Participants. The subjects were divided into three age groups: young (21–40 years old), middle (41–60 years old), and older (>60 years old).

### High-Resolution OCT, Imaging Protocol, Motion Correction, and Volume Fusion

Imaging was performed with a high-resolution SD-OCT (∼2.7-µm axial resolution, 128-kHz A-scan rate, ∼840-nm center wavelength) developed at MIT. Details on the specifications and instrument design are described in a prior publication.^16^ An orthogonal raster scan imaging protocol covering 6 × 6 mm (500 × 500 A-scans) was utilized, providing an isotropic A-scan spacing of 12 µm. Six repeated volume raster scans were acquired with the fast scan direction alternating between horizontal and vertical directions. To attain a single volume with enhanced signal-to-noise ratios and feature visibility, volumetric data fusion was performed within the motion correction and volume reconstruction in OCT (MoReOCT) framework. Volume fusion was comprised of computational 3D motion correction,^17^ compensation of OCT signal level (illumination) variations,^18^ and merging. The OCT images were flattened to the Bruch's membrane using machine learning–based layer segmentation^21^ and displayed in an axially stretched aspect ratio using a linear scale in order to preserve resolution. Conventional OCT images are displayed on a logarithmic scale which enhances dynamic range.

### Sub-Band Assessment: A-Scan Analysis

The sub-band located posterior to the ELM is axially thin and has low image contrast; therefore, we independently performed both qualitative (B-scan) analysis and quantitative (A-scan) analysis. All assessments were performed using ImageJ Fiji (National Institutes of Health, Bethesda, MD). The OCT images were stored in a 32-bit floating-point format, with an axial pixel size of 0.89 µm/pixel and a transverse pixel size of 12 µm/pixel.

To provide quantitative measure of the sub-band, the relative peak intensity ratio of the sub-band (%) was defined as the sub-band A-scan peak intensity divided by the ELM A-scan peak intensity (all in linear scale). The intensity of ELM was chosen as a reference because of its closer physical distance to the sub-band and its consistently identifiable OCT intensity, which was higher but on a similar scale to the sub-band intensity. The peak intensity ratio of the sub-band was computed at the distance points (0.5 mm, 1.0 mm, and 2.0 mm) from the foveal center determined by the ELM elevation. The axial position of the sub-band was measured using an A-scan at each distance point. The axial position relative to the ELM was measured from the axial center of the ELM peak to the axial center of the sub-band peak. If shadowing artifacts were present, a representative A-scan was chosen from ±5 neighboring A-scans (±60 µm lateral). When the sub-band was not visible on the B-scan, we utilized the median axial position of the sub-band peak (4.9 µm posterior to the ELM when the sub-band was visible) to measure its intensity.

### Sub-Band Assessment: B-Scan Grading

The ImageJ “Auto” function, under the “Brightness and Contrast” tool, utilizes the histogram of the image data and its minimum and maximum values to determine the image display parameters. The maximum image display parameter was automatically adjusted for each dataset by running the “Auto” function multiple times, such that the outer retinal bands (ELM to RPE–Bruch's membrane complex) were saturated and visually uniform between the subjects. Due to the weaker signal intensity of the sub-band feature, saturation of the outer retinal bands was necessary to improve and confirm its visibility. The ImageJ auto adjustment for image brightness and contrast is similar to how commercial instruments display images with varying signal-to-noise ratios.

**Figure 1.**
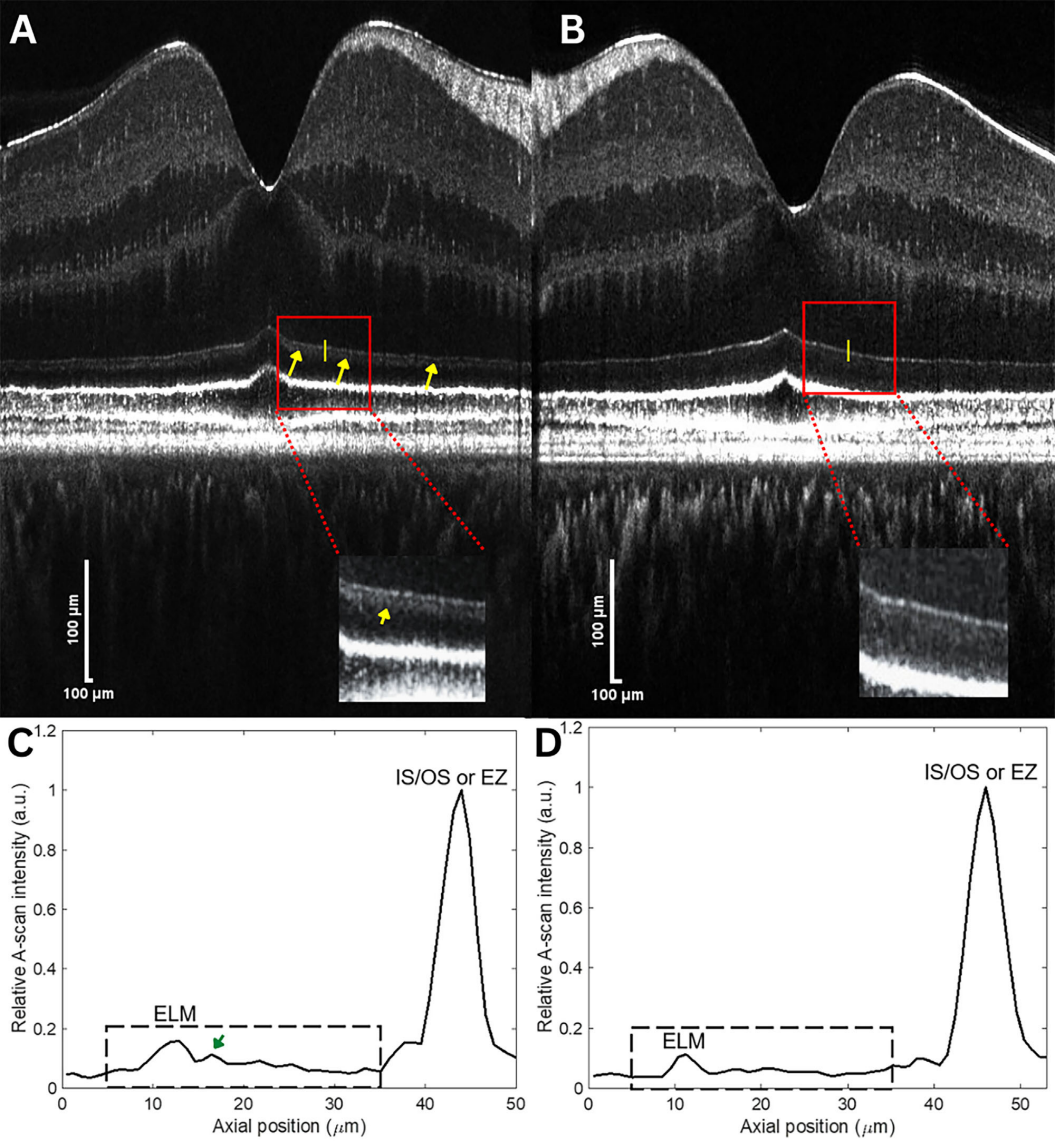
Representative high-resolution SD-OCT B-scans and corresponding A-scans. Six raster-scanned volumes (6 mm × 6 mm) were motion corrected and volume fused to increase feature visibility. Images extracted from the volume were axially stretched, flattened to Bruch's membrane, and displayed in linear scale to facilitate reader grading. (**A**, **B**) Representative B-scan showing a visible sub-band (*yellow arrows*) posterior to the external limiting membrane in a 57-year-old female subject (**A**) and not-visible sub-band in a 29-year-old female subject (**B**). The image contrast was adjusted to saturate the OCT signals of the outer retina. The zoomed in images with enhanced contrast provide a clear view of the sub-band. (**C**, **D**) Corresponding A-scans at *yellow lines* in (**A**) and (**B**), respectively. The *green arrow* in (**C**) indicates the A-scan peak of the sub-band.

Three OCT experts independently assessed the foveal center B-scans at each distance point (0.5 mm, 1.0 mm, and 2.0 mm) on both sides (nasal [N], temporal [T]) of the fovea and graded whether the sub-band posterior to the ELM was visible, not visible, or could not be determined (Fig. 1). The sub-band was denoted as ‘visible’, when there was a hyperreflective sub-band just posterior to the ELM. When the sub-band was visible, a hyporeflective gap separating the ELM and the sub-band could also be visualized. All graders were trained using representative high-resolution OCT images prior to grading. The final grading to indicate visible or not-visible sub-bands was determined by the majority reading results (two or more graders, out of three graders). When the sub-bands were graded as “cannot be determined,” open adjudication between the graders was performed to determine the final grading.

### Statistical Methods

The data were analyzed using SPSS Statistics 23.0 (IBM, Chicago, IL) to determine statistical significance. The relative peak intensity ratio of the sub-band to ELM at all distance points between the age groups was evaluated using the Kruskal–Wallis test, and its association with age was assessed using Spearman’s correlation coefficient without exclusion of any sample data. The Pearson χ^2^ test was used to evaluate differences in B-scan gradings across the age groups at all distance points. Cramér's *V* value was used to measure the strength of an association between two categorical variables, with a value close to 0 indicating no association and a value bigger than 0.25 suggesting a very strong relationship.^22^ The effect of DM status on the visibility was also analyzed using the Pearson χ^2^ test. Cohen's kappa (κ) was utilized to assess inter-reader agreement.^23^ Cohen's κ values can range from –1 to 1. A value of 1 indicates perfect agreement, 0 indicates agreement expected by chance, and negative values suggest systematic disagreement.

## Results

Forty-four eyes of 44 subjects with healthy maculas (age range, 21–90 years) were imaged and categorized into three age groups: young (mean ± SD, 29 ± 5 years; *n* = 13 eyes), middle (52 ± 6 years; *n* = 18 eyes), and older (70 ± 7 years; *n* = 13 eyes). Table 1 provides detailed demographic information.

**Table 1.** Demographics of Study Participants

Demographic	Value
Young Age Group (*n* = 13)	
Age (y)	
Mean ± SD	29 ± 1
Median (IQR)	29 (8)
Range	22–38
Male, *n* (%)	8 (61.5)
Race, *n* (%)	
White	8 (61.5)
Asian	4 (30.7)
Black	1 (7.6)
Eye, *n* (%)	
Right	4 (30.7)
Left	9 (69.3)
Middle Age Group (*n* = 18)
Age (y)	
Mean ± SD	52 (1)
Median (IQR)	52.5 (9)
Range	42–60
Male, *n* (%)	10 (55.5)
Race, *n* (%)	
White	8 (44.4)
Asian	1 (5.5)
Black	7 (38.8)
Unknown	2 (11.1)
Eye, *n* (%)	
Right	9 (50.0)
Left	9 (50.0)
Older Age Group (*n* = 13)
Age (y)	
Mean ± SD	70 (2)
Median (IQR)	68 (26)
Range	62–90
Male, *n* (%)	5 (38.4)
Race, *n* (%)	
White	9 (69.2)
Asian	2 (15.3)
Black	2 (15.3)
Eye, *n* (%)	
Right	7 (53.8)
Left	6 (46.2)

### Relative A-Scan Peak Intensity Ratio and Axial Position of the Sub-Band

Figure 1 shows representative B-scans with visible and not-visible sub-bands and their corresponding A-scans. The relative peak intensity ratios of the sub-band to the ELM in each group are shown in Table 2. When the sub-band was visible to readers on the B-scan, this corresponded to a 60% or greater relative intensity of the sub-band to the ELM on the A-scan. The means and SDs of the peak intensity ratios of the sub-bands in the young, middle, and older age groups were 71.6 ± 4.9%, 77.5 ± 2.3%, and 85.2 ± 4.2%, respectively. The sub-band was located 4.9 µm (median) posterior to the ELM when the sub-band was visible on B-scans (interquartile range [IQR], 4.5–5.6 µm). Table 2 also shows statistically significant differences among the age groups in the peak intensity ratio of the sub-band relative to the ELM at 0.5 mm N (*P* = 0.029) and 2 mm T (*P* = 0.001). All other distance points revealed non-significant statistical differences between age groups. The relative peak intensity ratio of the sub-band to ELM showed a positive correlation (Spearman correlation coefficient, *r*) with age at distance points 1 mm T (*r* = 0.375; *P* = 0.012) and 2 mm T (*r* = 0.511; *P* = 0.000) from the foveal center, suggesting increased visibility of the sub-band with age.

**Table 2.** A-Scan Intensity Ratios of the Sub-Bands Relative to the ELM and B-Scan Grading Across the Various Age Groups

	Distance From Foveal Center					
Age Groups	2 mm N	1 mm N	0.5 mm N	0.5 mm T	1 mm T	2 mm T
A-Scan Peak Intensity Ratios (%) of the Sub-Bands Relative to the ELM					
Age groups, mean ± SD
Young (*n* = 13)	79 ± 9	76 ± 18	65 ± 19	68 ± 14	69 ± 17	75 ± 12
Middle (*n* = 18)	78 ± 17	75 ± 16	80 ± 19	76 ± 17	75 ± 15	81 ± 11
Older (*n* = 13)	87 ± 10	86 ± 10	83 ± 11	78 ± 9	85 ± 15	92 ± 12
Kruskal–Wallis test for differences among age groups, *P*					
Age groups	0.056	0.082	0.029^*^	0.197	0.065	0.001^*^
Spearman correlation coefficient (*r*) with age (*P*)					
Age	0.256 (0.094)	0.162 (0.293)	0.283 (0.063)	0.070 (0.652)	0.375 (0.012)^*^	0.511 (0.000)^*^
B-Scan Grading Across Different Age Groups, Pearson χ^2^ (*P*)					
Age groups	6.70 (0.04)^*^	8.92 (0.01)^*^	3.74 (0.15)	5.02 (0.08)	12.62 (<0.01)^*^	13.10 (<0.01)^*^
Cramér's *V*	0.39	0.45	0.29	0.34	0.54	0.55

* *P* < 0.05.

### Visibility of the Sub-Band Based on B-Scan Grading

**Figure 2.**
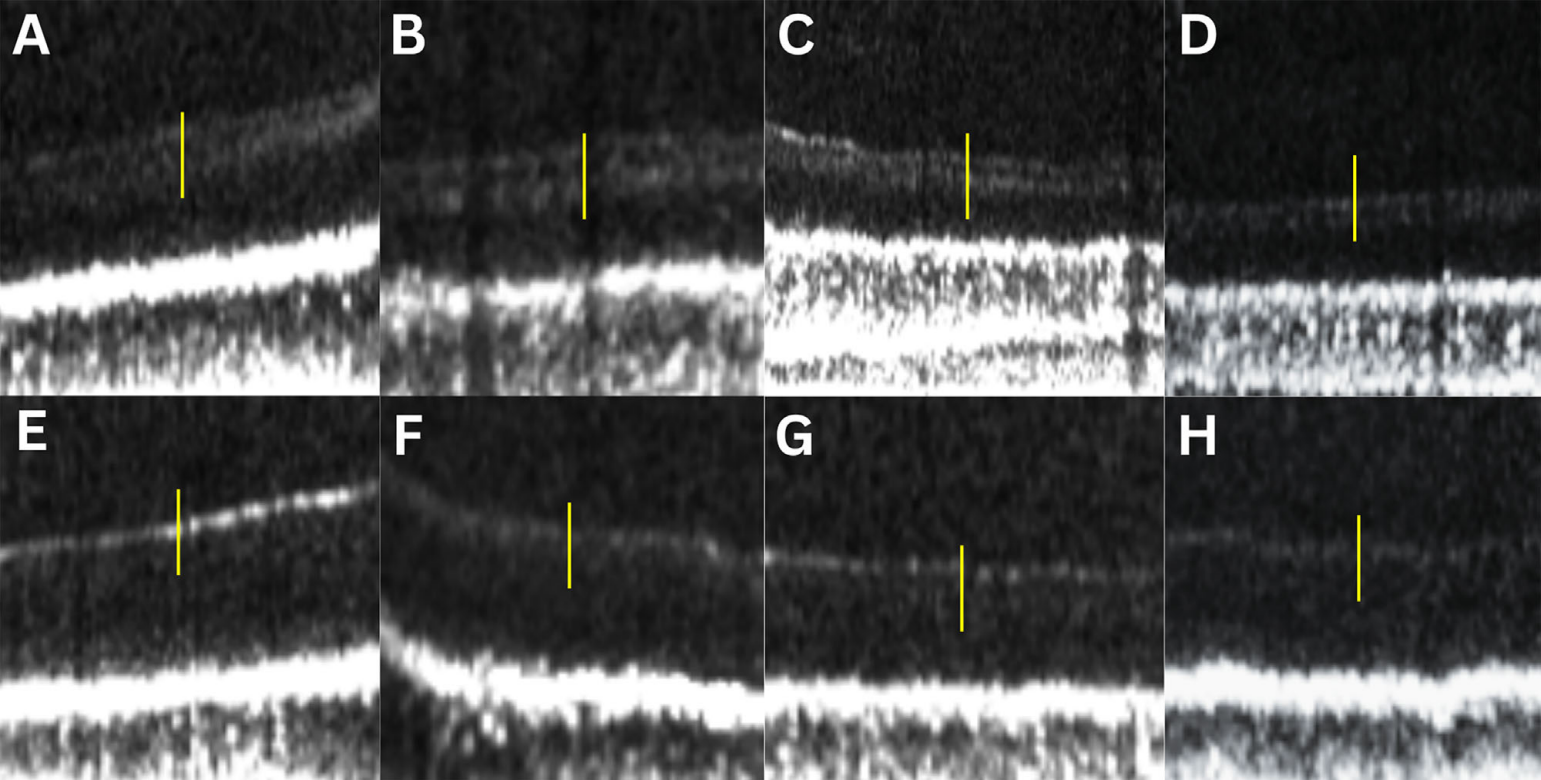
Contrast-enhanced, zoomed-in OCT B-scans showing visible and not-visible sub-bands in different age groups and at different distance points (*yellow line*) from the foveal center. (**A**–**D**) Visible sub-bands in a 55-year-old female subject at 0.5 mm N (**A**), 67-year-old female subject at 1 mm N (**B**), 67-year-old male subject at 1 mm T (**C**), and 72-year-old female subject at 2 mm T (**D**). (**E**–**H**) Not-visible sub-bands in 32-year-old male subject at 0.5 mm T (**E**), 72-year-old female subject at 0.5 mm N (**F**), 50-year-old female subject at 1 mm T (**G**), and 27-year-old female subject at 2 mm T (**H**).

**Figure 3.**
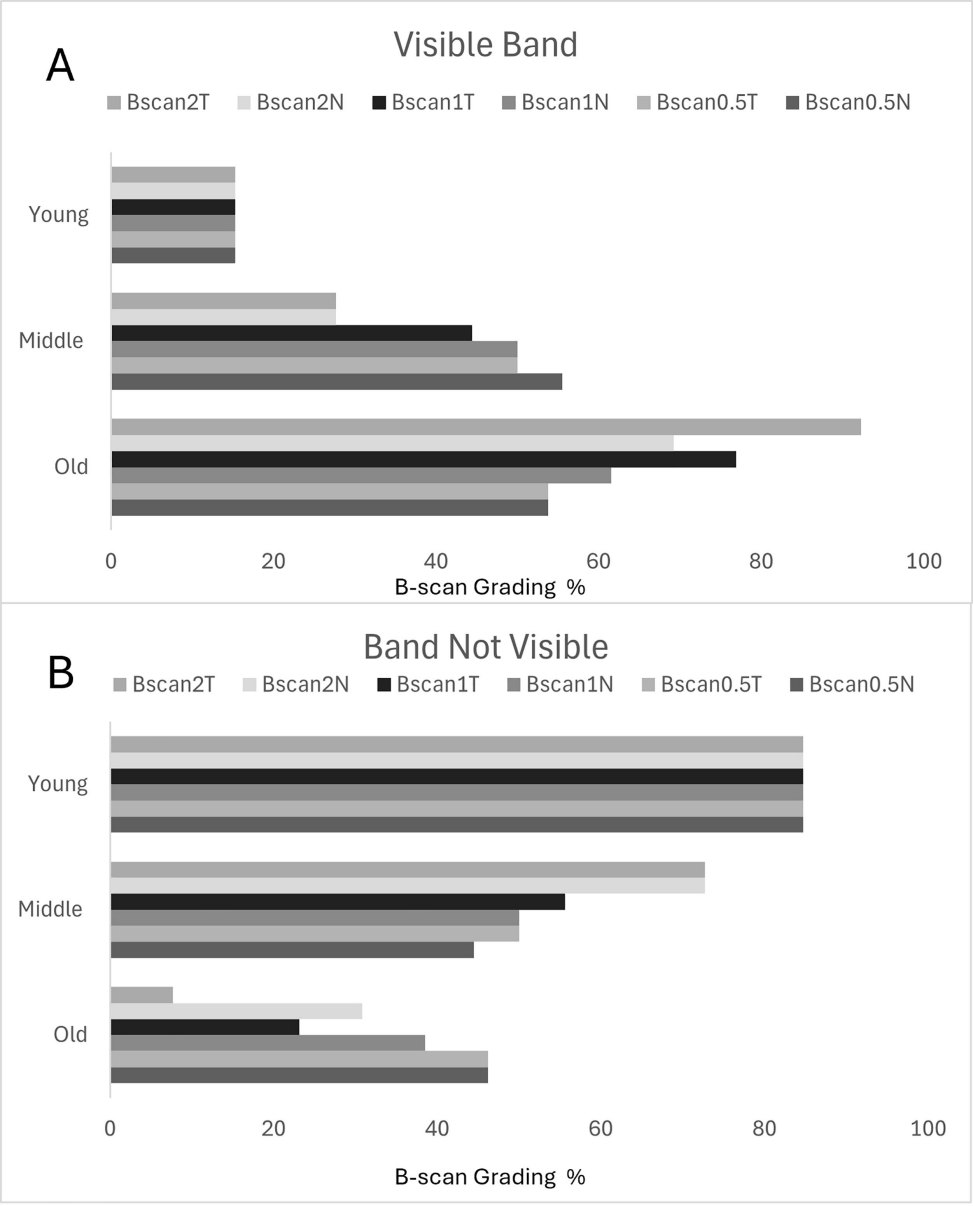
(**A**, **B**) Grouped bar charts representing B-scan grading results for visible sub-bands (**A**) and not-visible sub-bands (**B**) in each age group at all distance points.

Supplementary Table S1 shows B-scan grading results in each age group at all distance points. Figure 2 shows representative visible and not-visible sub-bands across the age groups at different distance points. The B-scan gradings across the distance points (0.5 mm, 1 mm, and 2 mm from the fovea) indicated visible sub-bands in 16.7%, 47.2%, and 66.7% of the young, middle, and older age groups, respectively. The sub-band was not visible in 83.3%, 52.8%, and 33.3% of the subjects in the young, middle, and older age groups, respectively. Figure 3 shows grouped bar charts representing varying visibility of the sub-band across distances in each age group.

A Pearson χ^2^ test for independence revealed a statistically significant association among age groups and B-scan gradings (χ^2^ > 3; *P* < 0.04) at all distance points, except at 0.5 mm N (*P* = 0.15) and 0.5 mm T (*P* = 0.08) (Table 2). The Cramér's *V* value for all distance points showed a very strong association (*V* > 0.25), suggesting a meaningful relationship between the age groups and B-scan gradings (Table 2). A Pearson χ^2^ test revealed no statistically significant association of the sub-band visibility with the status of DM in patients (Supplementary Table S2). In the young and middle age groups, the Fisher's exact test revealed significant differences between gradings at varying distance points. However, in the older age group, most gradings did not show statistical differences among the distance points (Supplementary Table S3).

### Inter-Reader Agreement for B-Scan Grading

Grader 1 and Grader 2 demonstrated fair to moderate levels of agreement, with κ coefficients of 0.43 for 0.5 mm N, 0.30 for 0.5 mm T, 0.24 for 1 mm N, 0.23 for 1 mm T, 0.15 for 2 mm N, and 0.12 for 2 mm T. When comparing Grader 1 with Grader 3, a fair level of agreement was observed, with κ coefficients ranging from 0.06 to 0.29 across different categories. The agreement between Grader 2 and Grader 3 demonstrated a higher level of consistency, with κ coefficients ranging from 0.53 to 0.69. The gradings near fovea showed greater agreement than those in the perifovea. Overall, the results suggest a fair to moderate level of agreement among the graders with some variability due to the qualitative and subjective nature of grading, particularly on newly identified features.

## Discussion

Multiple outer retinal bands have been revealed and investigated the last three decades since the advent of OCT, with some of their origins still being debated.^3^^–^^10^^,^^24^^,^^25^ Utilizing higher resolution OCT systems unlocks the potential to unveil finer and previously unrecognized features within the retina. In this study, the sub-band posterior to the ELM was investigated in normal eyes from different age groups using a high-resolution SD-OCT prototype at a 840-nm center wavelength. Instruments with comparable axial resolutions are now commercially available.^26^^,^^27^ Thus, larger scale clinical studies of new imaging features that can be visualized by high-resolution OCT are becoming possible.

The sub-band posterior to ELM was evaluated both qualitatively (B-scan grading) and quantitatively (A-scan analysis). We analyzed the sub-band using visibility (a subjective metric) as a qualitative measure, showing more analytical details and variability of this sub-band. Such qualitative measures are an important criterion for clinicians when studying a new OCT feature and help develop a quantitative metric with better correspondence to human perception. To our knowledge, this sub-band feature has not been previously revealed nor investigated using standard NIR OCT clinical instruments. Utilizing machine learning–based layer segmentation to flatten images to Bruch's membrane,^21^ extended bit-depth data, axial stretching, and linear scale display enables signals from the sub-band to be visualized, even though they are substantially weaker than the outer retinal bands. This points out the need to retain an extended bit depth, rather than using limited bit depth data, which is a standard in many commercial instruments. The standard methods in commercial instruments are designed to rapidly visualize larger scale retinal features, displayed using a single choice of parameters. With improvements in image resolution and greater interest in finer scale structures as possible markers of disease, different display and reading methods (flattened, axially stretched, in linear) are necessary to improve the visibility and gradability.^28^ Furthermore, the improved axial resolution of high-resolution SD-OCT combined with computational 3D motion correction and volume fusion allowed us to visualize fine details more clearly and delineate the sub-band more precisely in this study.

**Figure 4.**
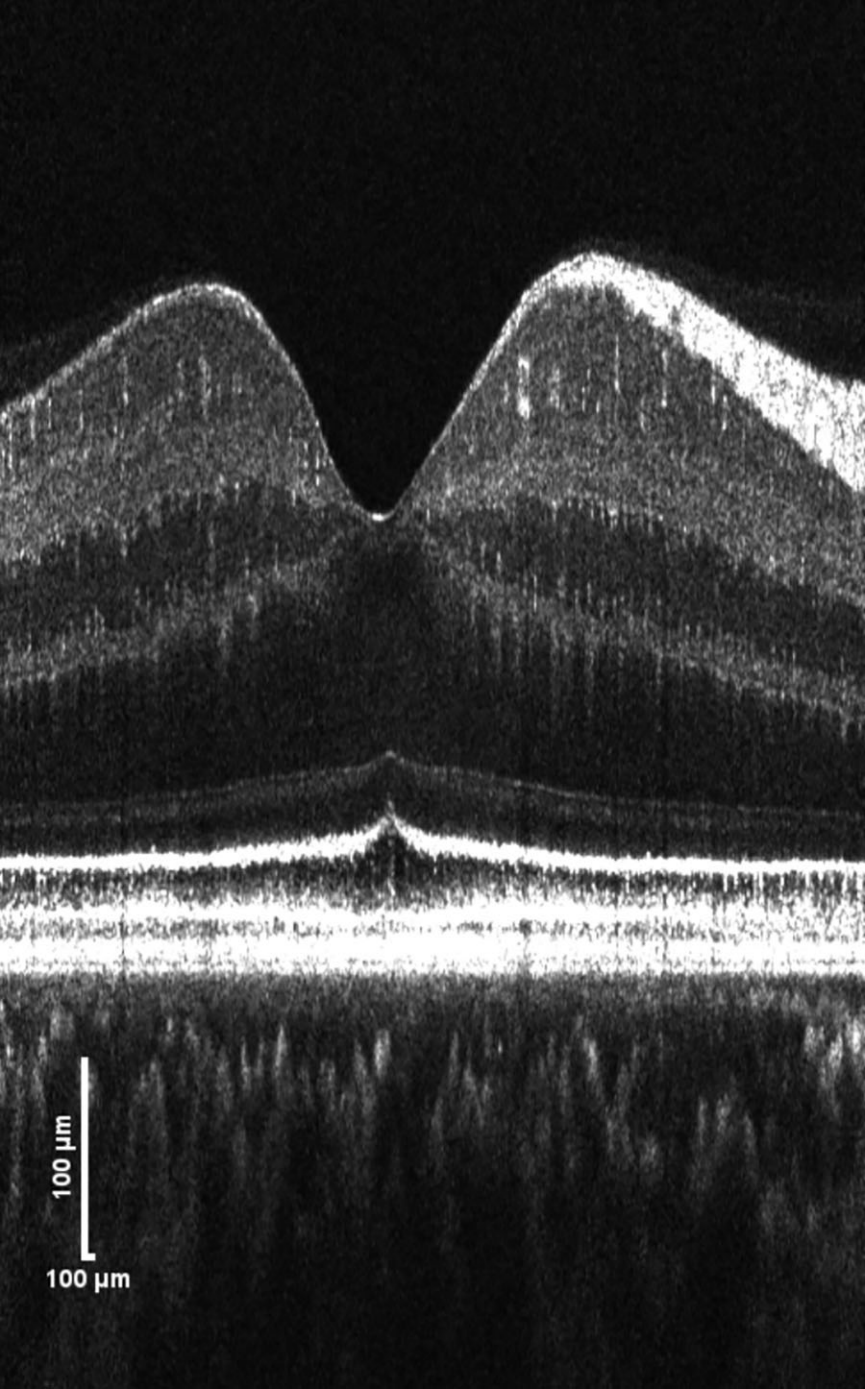
Representative high-resolution SD-OCT B-scan of a 57-year-old female subject showing that the sub-band in the foveola appears too thin or absent, whereas it is visible in the parafovea. Six raster-scanned volumes (6 mm × 6 mm) are motion corrected and volume fused to increase feature visibility. Images extracted from the volume are axially stretched, flattened to Bruch's membrane, and displayed in linear scale to facilitate reader grading.

Our observation is consistent with the work of Srinivasan et al.,^14^ who used a visible-light OCT prototype instrument and observed a subtle reflectivity division in the hyporeflective space between the ELM and IS/OS junction (or EZ), which they identified as myoid “m” (the sub-band referred to in this study, directly posterior to ELM) and inner ellipsoid “e” (space between the sub-band and IS/OS junction, or EZ, which is more hyporeflective than the sub-band). All eccentricities outside the foveola showed a typical pattern of myoid zone that was more reflective than the inner ellipsoid,^14^ consistent with our findings (Figs. 1, 2). In the foveola, the sub-band appeared too thin or absent (Fig. 4) for further analysis, but this finding agrees with the rapidly decreasing myoid length in the foveola (see figure S7 from Srinivasan et al.^14^ derived from the histologic study^29^). The visibility of the sub-band in our study may correspond to the presence of a myoid zone. However, note that the decreased visibility of the sub-band (or not-visible sub-band) in our study does not suggest the absence of a myoid zone because very subtle intensity differences between the ELM and IS/OS junction (or EZ) can still be observed. We suggest that NIR high-resolution OCT can also reveal the proposed myoid and inner ellipsoid zone and the visibility increases with normal aging.

Three age groups (young, middle, and older) were used to examine the sub-band and its age-associated changes. In general, increased visibility of the sub-band (myoid) with age was observed. The B-scan gradings showed increasing visibility of the sub-band in both nasal and temporal regions with older age groups. The A-scan analysis showed an increasing peak intensity ratio of the sub-band to the ELM with the older age group, which contributed to the improved sub-band visibility, and revealed a positive statistical correlation with age at 0.5 mm N, 1 mm T, and 2 mm T (Supplementary Table S3).

Such changes may be related to age-associated changes in photoreceptors^30^^–^^32^ and Müller cells which impact changes in OCT reflectivity, but determining the exact relationship in normal eyes would likely require corresponding histological assessment. Although we cannot validate the cause of such changes in this study, possible explanations from previous animal studies may suggest predominant rod photoreceptor degeneration with aging and mitochondrial swelling, a common occurrence in aging or degenerative tissues.^30^^,^^33^ In addition, Müller cell vulnerability with aging could have contributed to the changes in the visibility of the sub-band. Müller cells undergo oxidative damage with aging of the retina,^34^ causing age-related gliotic, lipid peroxidation, and edematous changes in their processes.^35^ Photoreceptors also undergo concurrent oxidative–nitrosative stress with aging.^35^

It is important to note that the ELM and sub-band may be observed as the thickened ELM under commercial NIR SD-OCT instruments. Thickening of the ELM has been previously described in early-onset Stargardt disease, hypothesized to be a protective response of Müller cells and interactions between photoreceptors and Müller cells;^36^^,^^37^ however, the spectrum of ELM thickening due to Stargardt disease is much wider and substantial compared to the sub-band discussed in this study.

This study advances our understanding of the hyporeflective space between the ELM and IS/OS junction (or EZ), which may correspond to the myoid and inner ellipsoid zone of the photoreceptor. This study uses a NIR high-resolution OCT prototype instrument combined with advanced 3D motion correction and volume-fusion methods, which improved layer visibility to enable examination of the sub-band. High-resolution commercial OCT instruments should also be able to visualize the sub-band; however, it is important to use extended bit depth OCT data viewed in linear scale, rather than conventional reduced bit depth OCT data viewed in logarithmic scale. In addition, modifications to the B-scan averaging algorithms used in commercial instruments may be required in order to reduce effects of feature blurring from eye motion. The signal-to-noise ratio may decrease with older age, which may reduce the relative sub-band intensity. The linear intensities used to compute the peak intensity ratio were not corrected for the noise. In the current study, however, the signal-to-noise ratio did not show significant differences between that in the visible and not-visible groups, suggesting that the visibility of the sub-band is not directly associated with the signal-to-noise ratio. Furthermore, averaging reduces noise, which makes image interpretation less susceptible to image degradation due to low signal strength.^18^

Limitations of this study include relatively small sample size, cross-sectional design, B-scan–based assessment, and lack of pathologic eyes. Future studies will include eyes with pathologies, such as age-related macular degeneration, retinitis pigmentosa, Stargardt diseases, and retinal vascular disorders. The development of an automated analysis tool will enable volumetric assessment of the sub-band. Although there was a higher correlation of the sub-band A-scan intensity with age in the temporal region, B-scan gradings showed significant differences of the sub-band visibility in both temporal and nasal regions. Volumetric analysis with larger cohorts would be necessary to investigate topographic dependence of this sub-band in the future. Longitudinal studies would be advantageous to confirm the observed age-related patterns and further investigate the biological and clinical relevance of the sub-band and its association with photoreceptor integrity and Müller cells.

## Supplementary Material

Supplement 1
