# Supplementary material for: High-Resolution OCT Reveals Age-Associated Variation in the Region Posterior to the External Limiting Membrane
Source: Transl Vis Sci Technol. 2025 Jan 15;14(1):16. doi: 10.1167/tvst.14.1.16 (PMC11741063; doi:10.1167/tvst.14.1.16)
Supplement: Supplement 1 [file tvst-14-1-16_s001.docx]

| **B-scan Grading across Different Age Groups** | | | | | | |
| --- | --- | --- | --- | --- | --- | --- |
| **Age groups** | **Distance from Foveal Center** | | | | | |
|  | **2 mm N** | **1 mm N** | **0.5 mm N** | **0.5 mm T** | **1 mm T** | **2 mm T** |
|  | B-scan Grading – graded as “visible”, n (%) | | | | | |
| Young (n=13) | 2 (15.4) | 2 (15.4) | 3 (23.1) | 2 (11.1) | 2 (15.4) | 2 (15.4) |
| Middle (n=18) | 5 (27.8) | 12 (66.7) | 10 (55.6) | 9 (50.0) | 8 (44.4) | 7 (38.9) |
| Old (n=13) | 8 (61.5) | 8 (61.5) | 7 (53.8) | 7 (38.9) | 11 (84.6) | 11 (84.6) |
|  | B-scan Grading – graded as “not visible”, n (%) | | | | | |
| Young (n=13) | 11 (84.6) | 11 (84.6) | 10 (76.9) | 11 (84.6) | 11 (84.6) | 11 (84.6) |
| Middle (n=18) | 13 (72.2) | 6 (33.3) | 8 (44.4) | 9 (50.0) | 10 (55.6) | 11 (61.1) |
| Old (n=13) | 5 (38.5) | 5 (38.5) | 6 (46.2) | 6 (46.2) | 2 (15.4) | 2 (15.38) |

**Supplementary Table 1:** B-scan grading results across different age groups at distance points from the foveal center [0.5 mm, 1 mm, and 2 mm nasal (N) and temporal (T)].

| **Pearson Chi-Square test (p-values) for association of sub-band visibility on B-Scan with DM status** | | | | | | |
| --- | --- | --- | --- | --- | --- | --- |
|  | **2 mm N** | **1 mm N** | **0.5 mm N** | **0.5 mm T** | **1 mm T** | **2 mm T** |
| B-scan gradings | 0.445 | 0.911 | 0.495 | 0.104 | 0.614 | 0.973 |

**Supplementary Table 2:** Pearson Chi-Square test shows not significant association of sub-band visibility with diabetes mellitus (DM) status in patients (healthy patients or patients with DM without clinically diagnosed retinopathy).

| **Fisher’s Exact test (p-values) comparing gradings at 2 distance points from the fovea in each age group** | | | | | | |
| --- | --- | --- | --- | --- | --- | --- |
| Young  (n= 13) |  | **1 mm N** | **2 mm N** |  | **1 mm T** | **2 mm T** |
|  | **0.5 mm N** | 0.038* | 0.038* | **0.5 mm T** | 0.013* | 0.013* |
|  | **1 mm N** | **-** | 0.013* | **1 mm T** | **-** | 0.013* |
| Middle  (n= 18) |  | **1 mm N** | **2 mm N** |  | **1 mm T** | **2 mm T** |
|  | **0.5 mm N** | 0.043* | 0.036* | **0.5 mm T** | 0.015* | 0.0498* |
|  | **1 mm N** | **-** | 0.11 | **1 mm T** | **-** | 0.013* |
| Old  (n= 13) |  | **1 mm N** | **2 mm N** |  | **1 mm T** | **2 mm T** |
|  | **0.5 mm N** | 0.10 | 0.10 | **0.5 mm T** | 0.19 | 0.19 |
|  | **1 mm N** | **-** | 0.032* | **1 mm T** | **-** | 0.29 |

**Supplementary Table 3:** B-scan gradings at two different distance points (0.5 mm, 1 mm, and 2 mm nasal (N) and temporal (T)) in each group were compared using the Fisher’s Exact test. * denotes p-values < 0.05.
